# Eliciting maize defense pathways aboveground attracts belowground biocontrol agents

**DOI:** 10.1038/srep36484

**Published:** 2016-11-04

**Authors:** Camila Cramer Filgueiras, Denis S. Willett, Ramom Vasconcelos Pereira, Alcides Moino Junior, Martin Pareja, Larry W. Duncan

**Affiliations:** 1 Universidade Federal de Lavras, Department of Entomology, Lavras, 37200-000, Brazil; 2 Agricultural Research Service, United States Department of Agriculture, Center for Medical, Agricultural and Veterinary Entomology, Gainesville, 32608, USA; 3 Instituto de Biologia, Universidade Estadual de Campinas - UNICAMP, Departamento de Biologia Animal, Campinas, 13083-970, Brazil; 4 Citrus Research and Education Center, University of Florida, Department of Entomology and Nematology, Lake Alfred, 33850, USA

## Abstract

Plant defense pathways mediate multitrophic interactions above and belowground. Understanding the effects of these pathways on pests and natural enemies above and belowground holds great potential for designing effective control strategies. Here we investigate the effects of aboveground stimulation of plant defense pathways on the interactions between corn, the aboveground herbivore adult *Diabrotica speciosa*, the belowground herbivore larval *D. speciosa*, and the subterranean ento-mopathogenic nematode natural enemy *Heterorhabditis amazonensis*. We show that adult *D. speciosa* recruit to aboveground herbivory and methyl salicylate treatment, that larval *D. speciosa* are relatively indiscriminate, and that *H. amazonensis* en-tomopathogenic nematodes recruit to corn fed upon by adult *D. speciosa*. These results suggest that entomopathogenicnematodes belowground can be highly attuned to changes in the aboveground parts of plants and that biological control can be enhanced with induced plant defense in this and similar systems.

Plants simultaneously inhabit two dynamic environments. In addition to procuring energy through photosynthesis, plant shoots must contend with a variety of aboveground herbivores. Likewise, in addition to procuring water and nutrients, plant roots must contend with attacks from below. Attacks against plant roots and shoots have driven evolution of plant defenses that protect the plant both above^1^ and belowground^2^,^3^,^4^.

Damage to the plant, above or belowground by herbivore or pathogen, can induce specific plant responses mediated by defense pathways within the plant. These pathways are myriad, interrelated, and diverse, but two prominent pathways are the salicylic acid pathway and the jasmonic acid pathway^5^. The salicylic acid pathway, mediated predominantly by the plant hormone salicylic acid, is thought to be induced by and primarily responsible for defense against pathogens feeding on living tissue^6^. In contrast, the jasmonic acid pathway, mediated predominantly by jasmonic acid, is thought to be induced by and predominantly responsible for defense against herbivores^6^. These defensive pathways are not independent; there can be antagonistic cross-talk between the salicylic and jasmonic acid pathway^5^. While these plant defensive pathways may be directly induced through pathogen infection or herbivory, similar defense responses may be induced by volatile organic compounds produced by insects or other plants^7^ or through application of methyl salicylate or methyl jasmonate elicitors^8^,^9^.

Products produced after induction of these pathways can confer increased resistance to pathogens and herbivores^5^ by altering plant palatability^10^ or recruiting natural enemies through release of volatile organic compounds^11^,^12^. Attraction of natural enemies through release of herbivore induced plant volatiles is a well known phenomenon both aboveground^13^,^14^ and belowground^15^,^16^ where entomopathogenic nematodes recruit to herbivore induced cues and infect insect larvae feeding on plant roots. In citrus, for example, feeding by the weevil *Diaprepes abbreviatus* induces release of pregeijerene which recruits an array of entomopathogenic nematodes^16^,^17^. Similarly, in corn, feeding by the beetle *Diabrotica virgifera virgifera* induces release of E-*β* caryophyllene which recruits the entomopathogenic nematode *Heterorhabditis megidis*^15^.

The effect of root herbivory on release of herbivore induced plant volatiles is not limited to the rhizosphere, however. Belowground herbivory can induce defenses aboveground^3^, while herbivory aboveground can have consequences for belowground herbivores^18^. These effects need not be reciprocal or equivalent; in some cases, belowground herbivory can stimulate responses in both the root and the shoot, while aboveground herbivory elicits shoot only responses^19^.

The role for plant defense pathways in mediating tritrophic responses to pests and natural enemies both above and belowground has been little explored, particularly in regard to behavioral effects of defense induction at higher trophic levels. To explore these interactions, we investigate the effect of aboveground induction of plant defense pathways in corn (*Zea mays L*.) on recruitment of adults of the corn rootworm pest *Diabrotica speciosa* (Germar) (Coleoptera: Chrysomelidae) aboveground, recruitment of larval *D. speciosa* belowground, and recruitment of the entomopathogenic nematode *Heterorhabditis amazonensis* belowground (Fig. 1).

While the adult beetle *D. speciosa* is a widespread, prominent, and polyphagous pest of corn and soybeans in South America, much of the damage to corn results from *D. speciosa* larvae feeding on corn roots^20^,^21^. *H. amazonensis* is a natural enemy of *D. speciosa* present endemically and sometimes applied for biological control^22^,^23^. Corn, *D. speciosa*, and *H. amazonensis* constitute a representative model system; the effects of plant defense pathway stimulation on the above and belowground multitrophic interactions present in this system hold ramifications and implications not only for our understanding of such systems generally, but also for designing intelligent biological control strategies in the field.

## Results

To investigate the effect of aboveground induction of plant defensive pathways in corn on recruitment of pests and natural enemies above and belowground, we presented undamaged, mechanically damaged, pest damaged, and elicitor treated plants to adult beetle *D. speciosa*, larval *D, speciosa*, and *H. amazonensis* entomopathogenic nematodes in multiple-choice and two-choice olfactometers.

### Adult *D. speciosa* Response

To determine effects of plant defense induction on pest behavior aboveground, adult *D. speciosa* were presented with combinations of undamaged, mechanically damaged, pest damaged, and elicitor treated plants in two-choice, Y-tube olfactometers. Pest damaged plants had been fed upon aboveground by five adult *D. speciosa* for 48 hours prior to the start of the experiment. Elicitor treated plants received foliar applications of solutions containing methyl salicylate or methyl jasmonate; soils and roots did not receive treatment. Responses to treatment combinations were monitored, as was the time spent in making the choice with the idea that preference would be revealed not only by choosing a particular treatment, but also faster response times to that treatment.

Indeed, multivariate analysis of variance revealed that treatment had a significant (*F* = 2.63; *df* = 15,146; *P* = 0.002) effect on adult *D. speciosa* preference and response time in Y-tube bioassays. Data conformed to assumptions of normality (visual inspection; *P* > 0.05, Shapiro Wilk Test) and homoscedasticity (*P* = 0.18, Levene’s Test). The linear combination of preference and response time identified by MANOVA and used as the *Diabrotica* Time Preference Index is


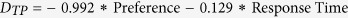


**Figure 2:**
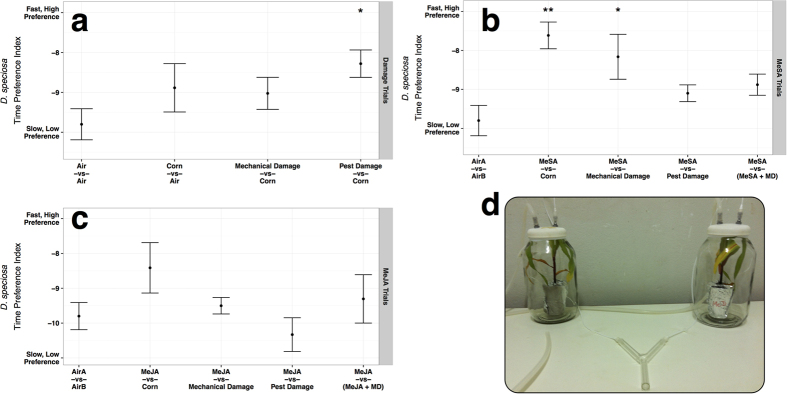
Adult *D. speciosa* responses in two-choice, Y-tube bioassays. (**a**) Responses to undamaged, mechanically damaged (MD) and pest damaged (fed upon by other adult *D. speciosa* corn seedlings. (**b**) Responses to Methyl Salicylate (MeSA) treated corn seedlings. (**c**) Responses to Methyl Jasmonate (MeJA) treated corn seedlings. (**d**) Y-tube apparatus used for assays. Clean humidified air is pumped into the chambers containing treatments then subsequently into the Y-tube. Adult beetles are released in the bottom of the Y and allowed to choose between treatments. *D. speciosa* Time Preference index is constructed from average response times and preferences for the top treatment in a pair (i.e. for Corn in Corn vs Air); higher numbers indicate a faster response and a greater preference for the top treatment in a pair. Points and error bars denote mean *D. speciosa* response (n = 10) and standard error respectively. A single asterisk denotes significant differences (P < 0.05) in response as compared with a baseline of Air vs Air. Double asterisks denote significance at P < 0.01.

With this index, adult *D. speciosa* demonstrated a trend to preferring higher levels of damage; corn fed upon by other adult *D. speciosa* significantly (*P* = 0.045, Dunnett’s Test) recruited more adult *D. speciosa*, faster (Fig. 2a). Interestingly, methyl salicylate (MeSA) treated corn seedlings recruited more adult *D. speciosa*, faster when contrasted with undamaged (*P* = 0.001, Dunnett’s Test) and mechanically damaged corn seedlings (*P* = 0.01, Dunnett’s Test) (Fig. 2b). Contrasts with methyl salicylate treated plants versus pest damaged plants and methyl salicylate treated mechanically damaged plants seemed to negate that effect (*P* > 0.17, Dunnett’s Test). Likewise, methyl jasmonate (MeJA) contrasts did not elicit significant effects (*P* > 0.14, Dunnett’s Test; Fig. 2c). The preference and faster response of adult *D. speciosa* to pest damaged and methyl salicylate treated corn seedlings in y-tube olfactometers (Fig. 2d) suggests that these adult beetles are attuned to volatile changes in the host plant. This similarity could indicate similarities in plant defense pathway induction between *D. speciosa* feeding and induction of the salicylic acid pathway. It appears that adult beetle feeding and methyl salicylate application elicit similar behavioral responses in recruitment of other adult *D. speciosa*.

### Larval *D. speciosa* Response

To determine effects of aboveground plant defense induction on pest behavior belowground, larval *D. speciosa* were presented with combinations of elicitors, undamaged, pest damaged, and elicitor treated plants in two-choice, sand-filled olfactometers.

**Figure 3:**
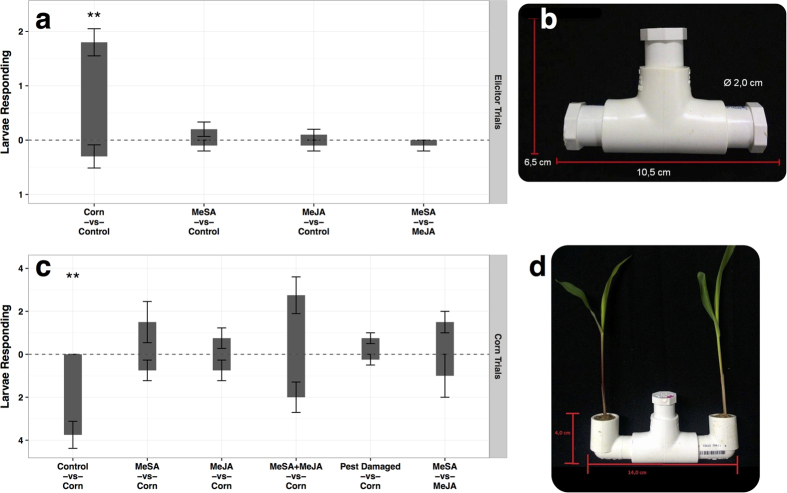
Larva *D. speciosa* responses in two-choice bioassays. (**a**) Responses to elicitor solutions placed on filter paper in opposing ends of olfactometer. (**b**) Inverted-T olfactometer used in (**a**). Elicitor solutions are placed on filter paper in opposing end caps of the inverted-T. Five larvae are released into the center. (**c**) Responses to corn seedlings treated aboveground with elicitor solutions. (**d**) Olfactometer used in (**c**). Treated plants are placed in opposing elbows; five larvae are released into the center. Larval responses above zero indicate preference for the top treatment in a pair (i.e. for Corn in Corn vs Control). Bars and error bars denote mean number of respondents (n = 10 Elicitor Trials; n = 4 Corn Trials) and standard error respectively. Double asterisks denote significance at P < 0.01.

To first test whether larval *D. speciosa* would recruit to the elicitors themselves, solutions containing either methyl salicylate (MeSA) or methyl jasmonate (MeJA) were placed on filter paper in opposing ends of an inverted-T olfactometer. Larvae were released into the center then, after 24 hours, responding larvae were removed from the end caps and counted. Larval *D. speciosa* significantly (*P* = 0.002, t-test) preferred germinated corn seeds (positive control), but did not significantly (at α = 0.05) respond to elicitor solutions (Fig. 3).

To test whether larval *D. speciosa* would recruit to corn seedlings in which defense pathways had been induced, *D. speciosa* larvae were presented with treated seedlings placed in opposing ends of a two-choice olfactometer. Treated seedlings received foliar applications of elicitor solutions containing either methyl salicylate (MeSA) or methyl jasmonate (MeJA) while pest damaged seedlings were fed upon aboveground by three adult *D. speciosa* for twenty-four hours prior to the start of the experiment. While larval *D. speciosa* recruited readily to corn seedlings versus blank controls (*P* = 0.009, t-test), elicitor treated and pest damaged plants did not significantly (at α = 0.05) recruit additional larvae (Fig. 3).

The response of larval *D. speciosa* to corn seedlings suggests that, while larvae can detect and recruit to the presence of a food source (corn roots), induction of plant defense pathways aboveground, whether by herbivory or elicitor application, have little effect on larval behavior. This could potentially be due to a lack of plant defense pathway mediated changes belowground on the part of the plant, or due to an inability to detect and respond on the part of the larvae.

### Entomopathogenic Nematode *H. amazonensis* Response

**Figure 4:**
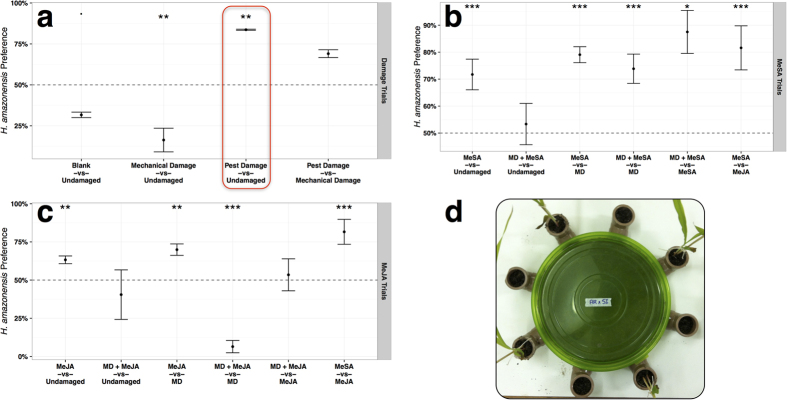
Entomopathogenic nematode *H.amazonensis* infective juvenile responses in multiple choice olfactometers. (**a**) Responses to undamaged, mechanically damaged (MD), pest damaged, and elicitor treated corn seedlings. Pest damaged corn seedlings had been fed upon aboveground by adult *D. speciosa*. Elicitor treated corn seedlings were treated with foliar applications of either methyl salicylate (MeSA) or methyl jasmonate (MeJA). Red box denotes novel finding of entomopathogenic nematodes recruiting to plants damaged by adult (i.e. non-larval) hosts. (**b**) Responses to Methyl Salicylate (MeSA) treated corn seedlings. (**c**) Responses to Methyl Jasmonate (MeJA) treated corn seedlings. (**d**) Multiple-choice olfactometer used for assays. Treatments are placed in peripheral elbows and entomopathogenic nematode infective juveniles released in the center. *H. amazonensis* preference is the percent infective juveniles responding to the top treatment in a pair (i.e. Pest Damage in Pest Damage vs Undamaged). Points and error bars denote mean percent response (n = 4) and standard error respectively. A period denotes marginal significance at P < 0.1; a single asterisk denotes significant differences at P < 0.05; double asterisks denote significance at P < 0.01; triple asterisks denote significance at P < 0.001.

To further explore the effects of aboveground plant defense induction belowground, we examined behavior of infective juveniles of the entomopathogenic nematode *H. amazonensis* in sand-filled multiple choice olfactometers (Fig. 4). These natural enemies, which hold great potential for biological control, were presented with combinations of undamaged, mechanically damaged, pest damaged, and elicitor treated plants. Pest damaged plants were fed upon aboveground for 48 hours by five adult *D. speciosa*; elicitor treated plants received foliar applications of solutions containing methyl salicylate (MeSA) or methyl jasmonate (MeJA).

Linear models with analysis of variance revealed significant effects of treatment (*P* ≤ 0.01 for all trials) on entomopathogenic nematode response. Data conformed to assumptions of normality (visual inspection; *P* > 0.05 Shapiro Wilk Test) and homoscedasticity (*P* > 0.05, Levene’s Test). Entomopathogenic nematode *H. amazonensis* infective juveniles significantly (P = 0.003, Dunnett’s Test) preferred corn seedlings fed upon aboveground by adult *D. speciosa* over undamaged control seedlings (Fig 4a). In contrast, mechanically damaged corn seedlings significantly (P = 0.009, Dunnett’s Test) repelled *H. amazonensis* infective juveniles. *H. amazonensis* infective juveniles displayed a marginally significant (P = 0.07, Dunnett’s Test) preference for undamaged corn seedlings versus blank controls.

Corn seedlings receiving foliar methyl salicylate (MeSA) treatment were significantly (P < 0.0004, Dunnett’s Test) more attractive to *H. amazonensis* infective juveniles belowground, except when combined with mechanical damage treatment (Fig 4b). Similarly, corn seedlings receiving foliar methyl jasmonate (MeJA) were significantly (P < 0.008, Dunnett’s Test) more attractive to *H. amazonensis* infective juveniles belowground, except in combination with mechanical damage treatments (Fig. 4c).

Combined, theses results suggest that the entomopathogenic nematode *H. amazonensis* is highly attuned to differences in status of corn plants upon which their insect hosts feed; they recruit to herbivorized plants and can demonstrate differential responses to mechanically damaged and pest damaged seedlings. Interestingly, stimulation of plant defense pathways through foliar elicitor application seems to mimic much of the same effects suggesting similarities between herbivory by adult *D. speciosa* and induction of plant defense pathways.

### Multitrophic Implications

Taken together, these results suggest a role for plant defense pathways in mediating the interactions between plants, herbivores, and natural enemies above and belowground. Indeed, while adult *D. speciosa* are somewhat responsive to herbivory and induction of plant defense pathways, *H. amazonensis* entomopathogenic nematodes belowground seem to be particularly attuned to aboveground induction of plant defense pathways (Table 1). In contrast, larvae do not seem to discriminate or respond to elicitor treatments in our assays (Table 1).

## Discussion

The effects of plant defense pathway stimulation aboveground on herbivore and natural enemy behavior above and belowground has implications for understanding and managing the interactions between corn, *D. speciosa*, and *H. amazonensis* as well as similar interactions in other systems. The ability of plant defense pathway stimulation aboveground, whether through herbivory by adult *D. speciosa* or methyl salicylate application, to recruit adult *D. speciosa* aboveground suggests a role for plant defense pathways in mediating adult distributions in the field. Adult *D. speciosa* are well known to monitor and respond to plant volatiles in the lab and in the field^24^,^25^, responding particularly well to cucurbitacins which may influence progeny fitness^26^. In our case, herbivory and stimulation of the salicylic acid pathway may induce release of compounds recognized as favorable by the adult *D. speciosa*. Such recognition may signal presence of other individuals, more available resources, or reduced plant defenses. Adult *D. speciosa* likely recruit to volatiles released by the plant to take advantage of such conditions. Indeed, observations from collections in the field support that hypothesis; adult *D. speciosa* are seldom found individually on corn plants in the field. Corn plants typically host many adult *D. speciosa*, most of them mating pairs. Volatiles emitted by prior feeding or stimulation of plant defense pathways may contribute to aggregation and mate finding in adult *D. speciosa*.

Interestingly, stimulation of plant defense pathways aboveground seems to have little effect on larval *D. speciosa* behavior belowground. This observation could be a boon for strategies using induced plant defenses to augment biological control in conjunction with other strategies to repel *D. speciosa* larvae^27^. Because larvae do not preferentially respond to elicitor treated plants, induced plant defense will likely not attract belowground larval pest populations in the field while augmenting control through recruitment of entomopathogenic nematode natural enemies.

Our results suggest that entomopathogenic nematodes can respond to stimulation of plant defense pathways aboveground through herbivory or elicitor application. Likewise, because *H. amazonensis* does not recruit to mechanially damaged plants, our results suggest that mechanical damage alone is not sufficient to stimulate a plant defense response. Indeed, monitoring of plant defense pathway stimulation by aboveground herbivory on the part of the entomopathogenic nematode *H. amazonensis* may hold adaptive significance; feeding aboveground by the adult *D. speciosa* indicates a greater likelihood of eventually finding suitable larval hosts, especially for cruiser nematodes like *H. amazonensis* which can travel large distances belowground in search of food^28^,^29^. As adult *D. speciosa* recruit to herbivorized plants where they are likely to encounter other members of their species, they are apt to mate and reproduce on the same plant, letting the eggs fall to soil in the immediate area. After hatching, the larvae seem non-discriminatory and will recruit to the nearest plant root. Entomopathogenic nematodes seem to have evolved to take advantage of this system, responding to the decision makers aboveground - the adult *D. speciosa* - in the search for food.

This search for food on the part of entomopathogenic nematodes can be augmented in this and similar systems. Aboveground, elicitor application can be considered as a means of influencing *D. speciosa* distributions. Belowground, aboveground application of elicitors may also be considered for influencing distribution of entomopathogenic nematodes. Additionally, the volatiles involved in mediating entomopathogenic nematode attraction to aboveground herbivory can be used to augment biological control of larval *D. speciosa* belowground. We are exploring that possibility.

## Methods

### Plant Materials

Bt transformed Herculex I (Dow AgroSciences, Pioneer Hi-Bred International) corn seedlings expressing the Cry1F gene were used in all experiments. This variety was developed primarily to control Fall Armyworm (*Spodoptera frugiperda*) and is in widespread use in Brazil. Seeds were germinated in moist vermiculite, then grown for twenty days in organic substrate. Prior to use in belowground bioassays with larval *D. speciosa* and entomopathogenic nematodes, corn seedling roots were gently washed to remove substrate before placement in olfactometers.

### Insect Rearing

Adult *D. speciosa* were collected from corn fields maintained by the Federal University of Lavras (Lavras, MG, Brazil) and taken directly to the laboratory for rearing following previously established methodology^30^. Adults were maintained on bean leaves (*Phaseolus vulgaris*) while eggs were collected from black gauze strips placed with the adults to induce oviposition. Eggs were washed from the gauze strips every two days then placed in petri dishes with moistened filter paper to maintain humidity until eclosion. Larvae were maintained on recently germinated corn seedlings in vermiculite until pupation. Eight days after eclosion, larvae were used in bioassays. Adult *D. speciosa* used in bioassays were taken directly from the field and used within a week of collection.

### Nematode Rearing

Entomopathogenic nematode *H. amazonensis* infective juveniles used in multiple choice olfactometers were obtained from cultures maintained at the Federal University of Lavras where the nematodes were reared on greater wax math (*Galleria mellonela*) larvae. Wax moth larvae were reared in the laboratory^31^ and maintained on artificial media^32^. When nematode infective juveniles were needed, *G. mellonela* larvae were inoculated with entomopathogenic nematodes^33^ and infective juveniles subsequently collected on White traps^34^. After collection, nematodes were maintained in culture flasks in aqueous suspension at 16 ± 1 °C and used in bioassays within a week of collection.

### Elicitor Preparation

For preparation of elicitors for application to the aboveground parts of maize seedlings, Tween 20 and ethanol were added to water such that final concentrations were 0.1 mL/L and 2.5 mL/L respectively. Concentrations of methyl salicylate and methyl jasmonate were adjusted to 0.5 mM then added to solution such that the amount used per plant per experiment was 65 μL and 109 μL respectively. Aliquots of 30 mL elicitor solution were applied to the aboveground foliage of corn seedlings using a spray bottle, a quantity sufficient for the corn seedling to become wet and to ensure homogeneous application. Contact of elicitor solution with the roots was prevented by an aluminum foil barrier.

### Adult *D. speciosa* Bioassays

The responses of adult *D. speciosa* to treated and untreated corn plants were evaluated in two-choice Y-tube glass olfactometers (Fig. 2d). Aboveground portions of plants with the desired treatments were placed in glass chambers where filtered, humidified air was introduced then pumped via teflon tubes to the olfactometer, one treatment per arm. Adult *D. speciosa* were introduced at the base of the Y-tube olfactometer and allowed five minutes to choose an arm; response time and treatment choice were monitored. Treatments were rotated every three insects to avoid positional effect. Ten replications of each treatment combination were conducted with each replicate consisting of ten insect choices. Clean glassware and new plants were used for each replicate.

Treatment combinations consisting of undamaged, mechanically damaged, pest damaged, and elicitor untreated and treated plants were used to determine the effect of plant defense pathway stimulation on *D. speciosa* response. Twenty day old corn seedlings were used in all experiments; undamaged corn seedlings were taken directly from the greenhouse 20 days after germination and immediately used in the experiment. Mechanically damaged corn seedlings were cut with a scalpel using a template replicating foliar damage by adult *D. speciosa* 48 hours prior to use in the experiment. Pest damaged plants were fed upon by five adult *D. speciosa* placed in mesh bags on the foliage of each plant 48 hours prior to use in the experiment. Elicitor treated (either methyl salicylate or methyl jasmonate) plants were treated 48 hours prior to use in experiments.

### Larval *D. speciosa* Bioassays

To examine larval *D. speciosa* responses belowground, two-choice sand filled olfactometers were used (Fig. 3b,d). Two choice olfactometers were constructed from 3/4 inch (1.9 cm) PVC pipe filled with washed autoclaved sand maintained at 12% moisture by volume. Larval responses were evaluated to treatments consisting of undamaged seedlings, damaged seedlings, elicitor treated seedlings, and elicitors.

For evaluating larval responses to volatile chemicals, treatments were applied to filter paper placed in the end caps of sand-filled, inverted T olfactometers (Fig. 3b). The above elicitor solutions were added in 30 ml aliquots to filter paper and allowed to dry. As above, controls consisted of the Tween 20 and ethanol solution without elicitors. Germinated corn seeds were used as positive controls. After placing filter paper or corn controls in the end caps of the sand-filled olfactometer, five 8 day old *D. speciosa* larvae were released into the center of the olfactometer. After an additional 24 hours, responding larvae were collected from the elbows and counted. Ten replications of each treatment combination were conducted.

For evaluating larval responses to elicitor treated plants, inverted olfactometers connected to PVC elbows were used (Fig. 3d). Seedlings were placed in opposing arms, appropriate treatments applied, and the olfactometer filled with sand. Insect damaged seedlings received three adult *D. speciosa* for twenty four hours prior to use in the experiment. For elicitor treated plants, elicitor solutions were applied to the aboveground parts of maize seedlings. Elicitor solutions were prepared as described above. Control plants (undamaged seedlings) received the Tween 20 and ethanol solution without elicitors. Twenty-four hours after application, five 8 day old *D. speciosa* larvae were released into the center of the olfactometer. After an additional 24 hours, responding larvae were collected from elbows and counted. Four replications of each treatment combination were conducted.

### Nematode Bioassays

Entomopathogenic nematode *H. amazonensis* response to treated and untreated corn plants was evaluated in sand-filled multiple-choice olfactometers consisting of a central chamber connected to eight arms into which corn seedlings were inserted (Fig. 4d). Olfactometers were constructed from 30 cm diameter plastic containers (Tupperware) perforated at equally spaced intervals to which eight 4 cm diameter PVC elbows were connected. Seventy two hours prior to the start of the experiment, olfactometers received corn seedlings and were filled with washed, autoclaved sand adjusted to 12% moisture by volume. After 72 hours of acclimation to the new environment, the aboveground foliage of corn seedlings was treated as described above to evaluate the ability of undamaged, mechanically damaged, pest damaged (above ground feeding by adult *D. speciosa*), and elicitor treated plants to recruit entomopathogenic nematodes belowground.

Treatment contrasts were arrayed around an olfactometer in an alternating pairwise fashion. For example, Blank vs. Undamaged contrasts were arranged as depicted in Fig. 4d. Four controls were alternated with four undamaged plants around the olfactometer for one replicate. Forty eight hours after treatment application, 2500 *H. amazonensis* infective juveniles were released in the center of each olfactometer. An additional 24 hours later, the olfactometers were disassembled, the responding nematodes extracted via Baermann funnel, and evaluated. Tests of known amount of nematodes placed in Baermann funnels and replicated 20 times yielded an extraction efficiency of 13.1 ± 1.4%. Nematode counts were adjusted accordingly; four replications of each treatment combination were evaluated.

### Statistical Analysis

Response times and preferences for adult *D. speciosa* in two-choice, Y-tube bioassays were analyzed using multivariate analysis of variance (MANOVA). Mean response time and proportion of adults responding to treatment of interest were calculated for each replicate; bioassay treatments were used to model variation in those two parameters. *Diabrotica* Time Preference Indices were constructed from the first linear discriminant function and evaluated with Roy’s greatest characteristic root test. Conformation to assumptions of normality and homoscedasticity was verified through visual examination of residual plots, the Shapiro-Wilk test, and Levene’s test. Significant results from MANOVA were further explored with Dunnett’s test (to avoid inflated Type I error), comparing treatments of interest to baseline adult *D. speciosa* responses to Air vs Air trials.

Larval *D. speciosa* responses in two-choice olfactometers were analyzed using one-sample t-tests after subtracting number of larvae responding to controls from responses to treatment of interest. Responses were examined for adherence to assumptions of normality by visual inspection with quantile-quantile plots and the Shapiro-Wilk test.

Nematode responses in eight-arm, sand-filled olfactometers were analyzed using linear models and analysis of variance with treatment and contrast (treatment combination) as factors. To avoid effects of aggregation (presented with equivalent treatments, nematodes will often aggregate in one treatment arm^35^,^36^,^37^, responses to individual treatments were summed within replicates. Assumptions of normality and heteroscedastity were examined by visual inspection of residual diagnostic plots, the Shapiro-Wilk test and Levene’s test. Post-hoc comparisons of nematode responses were conducted with Dunnett’s test to avoid α inflation.

All data were collated in Microsoft Excel then read into R version 3.2.2^38^ for analysis. RStudio version 0.99.484^39^ was used as a development environment. Various supplementary packages were used in R for additional functionality: xlsx^40^ for interface with Microsoft Excel, tidyr^41^ and dplyr^42^ and tidyr^43^ for data arrangement and summary statistics, ggplot2^44^ for graphics capabilities, car^45^ for MANOVA statistics, and multcomp^46^ and lsmeans^47^ for multiple comparisons using Dunnett’s test.

## Additional Information

**How to cite this article**: Filgueiras, C. C. *et al*. Eliciting maize defense pathways aboveground attracts belowground biocontrol agents. *Sci. Rep*. **6**, 36484; doi: 10.1038/srep36484 (2016).

**Publisher’s note:** Springer Nature remains neutral with regard to jurisdictional claims in published maps and institutional affiliations.

## Figures and Tables

**Figure 1:**
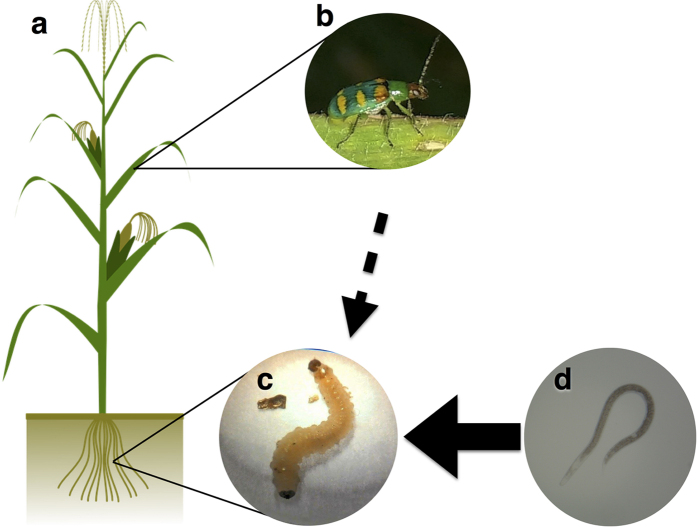
Multitrophic interactions in the *Diabrotica speciosa* system. Corn leaves in (**a**) are fed upon by adult *D. speciosa* in (**b**). Adult *D. speciosa* oviposit into the soil where larvae in (**c**) feed upon corn roots. *Heterorhabditis amazonensis* entomopathogenic nematode infective juveniles in (**d**) are subterranean natural enemies and parasitiods of *D. speciosa* larvae.

**Table 1:** Responses of herbivores (adult and larval *D. speciosa*) and natural enemies (the entomopathogenic nematode *H. amazonensis*) to undamaged, mechanically damaged, pest damaged (fed upon by adult *D. speciosa*) and elicitor treated corn seedlings.

	*Adult D. speciosa*	*Larval D. speciosa*	*H. amazonensis*
	Aboveground	Belowground	Belowground
Corn	ND	+	+
Mechanical Damage	ND	NA	−
Pest Damage	+	ND	+
MeSA	+	ND	+
MeJA	ND	ND	+

+indicates attraction; −indicates repellences; ND inducates no significant difference detected; NA indicates the combination was not assayed.
